# Ten years of Central IRB review for an NIH-funded large clinical trial network by an academic IRB: The NIH StrokeNet experience

**DOI:** 10.1017/cts.2025.10231

**Published:** 2026-01-02

**Authors:** Michael Linke, Angela Braggs-Brown, Susan K. Roll, Kareemah Mills, S. Iris Davis, Joseph P. Broderick, Pooja Khatri

**Affiliations:** 1 College of Medicine, Internal Medicine, University of Cincinnati, Cincinnati, OH, USA; 2 Human Research Protection Program, University of Cincinnati, Cincinnati, OH, USA; 3 College of Medicine, Neurology, University of Cincinnati, Cincinnati, OH, USA; 4 Neurology, Yale School of Medicine, New Haven, CT, USA

**Keywords:** Institutional Review Board, clinical trial network, reliance agreement, single IRB, human research protection program

## Abstract

In 2013, the National Institute of Neurological Disorders and Stroke established National Institutes of Health (NIH) StrokeNet to support multi-site clinical trials focused on stroke prevention, treatment, and recovery. The University of Cincinnati (UC) serves as the National Coordinating Center for StrokeNet. As part of the initiative, the UC StrokeNet Central IRB (SN-CIRB) was established at UC to serve as a single IRB to oversee StrokeNet trials. Since the SN-CIRB approved the first StrokeNet study in 2014, it has reviewed and approved 16 additional studies. Over this period, the UC Human Research Protection Program refined its review processes based on insights from earlier reviews. These improvements have improved efficiency while still ensuring the protection of study participants. The successful implementation and ongoing conduct of the SN-CIRB at UC demonstrate that an academic-based IRB can effectively serve as a Central IRB for a large clinical trial network.

## Background

The traditional approach of individual Institutional Review Board (IRB) oversight at each site in a multi-site clinical trial has long been considered inefficient and ineffective [1,2]. The National Cancer Institute (NCI) established the NCI Central IRB in 2001, streamlining the review and approval process for all NCI-sponsored Phase 3 treatment trials [3]. Building on this model, in 2011, the National Institute of Neurological Disorders and Stroke (NINDS) launched a Central IRB (CIRB) for the Network for Excellence in Neuroscience Clinical Trials (NeuroNEXT) [4]. In 2013, NINDS created National Institutes of Health (NIH) StrokeNet utilizing a CIRB to oversee multi-site clinical trials focused on stroke prevention, treatment, and recovery [5]. Since that time, several other clinical research networks have also successfully incorporated a single IRB (sIRB) process [6,7]. NIH and HHS mandated a sIRB review for multisite studies in 2018 and 2020, respectively. FDA issued a Notice of Proposed Rulemaking in 2022 that also proposed requiring sIRB requirements.

The StrokeNet infrastructure comprises 27 regional coordinating centers (RCCs) across the US, the National Coordinating Center (NCC) at the University of Cincinnati (UC), and a National Data Management and Statistical Center (NDMC) at the Medical University of South Carolina. Each StrokeNet study includes a Lead Study Site and Clinical Performance Sites (CPS). As part of this initiative, the UC StrokeNet CIRB (SN-CIRB) was established at the UC to serve as the sIRB for StrokeNet trials. Instead of establishing a new IRB, the UC IRB serves as the SN-CIRB and is administered through the UC Human Research Protection Program (UC HRPP). A dedicated IRB Chair oversees the SN-CIRB review of StrokeNet research studies.

The SN-CIRB oversees all aspects of IRB review, including initial study approvals, CPS approvals, study-wide and site-specific modifications, reportable events, and continuing reviews of NIH StrokeNet research. In collaboration with the NCC, the SN-CIRB executes reliance agreements with each CPS to ensure compliance with SN-CIRB requirements. The UC HRPP manages the SN-CIRB process electronically. The NCC, HRPP, and SN-CIRB continuously refine their processes based on experience and evolving guidance on sIRB operations, streamlining the ethical review process for multi-site stroke research. They strive to enhance efficiency while protecting the rights and welfare of participants. SN-CIRB is an example of how an IRB based at an academic health center can effectively operate as a sIRB for a large clinical research network.

To date, the SN-CIRB has approved 13 primary StrokeNet trials (Table 1). One of these is a multifaceted platform trial (STEP). The SN-CIRB has also approved five ancillary studies associated with the primary trials. Studies target acute stroke interventions to improve treatment responses, preventing the next stroke, and recovery or rehabilitation strategies to improve functional recovery post-stroke. The studies involve complex populations and settings, including vulnerable participants with cognitive impairment and minors. They include trials conducted in emergency departments where time-sensitive interventions add to the operational challenges. The SN-CIRB ensures appropriate expertise is applied to the ethical review of these studies, either through its standing board membership or by engaging ad hoc expert reviewers when specialized knowledge is required. For instance, studies involving minors were reviewed with input from pediatric experts at Cincinnati Children’s Hospital Medical Center.

**Table 1.** Primary StrokeNet studies approved by the UC Central IRB

Study	Submitted	Approved	Days to approval^†^	Sites approved^‡^	ClinicalTrials.gov ID
CREST 2*	8/9/2014	9/24/2014	46	69	NCT02089217
TELEREHAB*	1/26/2015	3/18/2015	51	53	NCT04657770
DEFUSE-3*	9/28/2015	10/14/2015	16	43	NCT02586415
ARCADIA*	2/21/2017	3/10/2017	17	173	NCT03192215
MOST*	2/5/2018	4/11/2018	65	99	NCT03735979
Sleep SMART	2/15/2018	4/11/2018	55	160	NCT03812653
TRANSPORT 2*	6/25/2018	9/12/2018	79	14	NCT03826030
SATURN	9/24/2018	11/28/2018	65	132	NCT03936361
I-ACQUIRE	12/4/2018	1/3/2019	30	17	NCT03910075
ASPIRE	2/3/2019	4/9/2019	65	188	NCT03907046
SISTER	4/12/2023	6/1/2023	50	49	NCT05948566
STEP	3/28/2024	4/25/2024	28	38	NCT06289985
TELEREHAB-2	9/25/2024	11/13/2024	49	3	NCT06682429

* Completed.

† Calendar days from initial submission to the UC-HRPP to final approval of the protocol by the SN-CIRB.

‡ As of June 30, 2025.

In 2020, the NINDS and NCC decided to expand the capacity for reviewing studies by adding an independent IRB to review several StrokeNet studies. SN-CIRB provides primary oversight of the NIH StrokeNet Standard Operating Procedures (SOPs) related to IRB operations, as described below, for managing the CIRB process for all StrokeNet studies.

## The SN-CIRB reliance agreement process

Relying institutions must document that they will rely on a reviewing IRB for oversight of human subject research conducted at their institutions, to ensure compliance with the regulations and to define the responsibilities of each institution. Typically, this is documented with a written reliance agreement signed by both parties. Historically, the reliance agreement process has been frustrating for reviewing IRBs and relying institutions. It has been overly complicated and often study-specific, leading to delays in the execution of agreements [8]. To alleviate some of these problems, the initial StrokeNet reliance agreement was based on NIH and OHRP templates available at the time, as well as the NeuroNext CIRB reliance agreement [4]. UC HRPP and the NCC collaborated to develop the SN-CIRB Reliance Agreement for the network. The RCCs reviewed the initial draft in coordination with their HRPPs, and revisions were made based on the feedback received. A second draft was circulated again for review by the RCCs before being finalized.

In 2014, the 25 StrokeNet Regional Coordinating Centers (RCC) executed the SN-CIRB Reliance Agreement. The median turnaround time for the RCCs to fully execute the agreement was 30 calendar days. The agreement was then initially executed by 161 StrokeNet performance sites. The median turnaround time for the initial sites to fully execute the agreement was 27 calendar days. Since then, over 800 research sites have executed the reliance agreement as additional studies have been approved.

In 2022, the StrokeNet NCC and UC HRPP determined that a new Reliance Agreement was needed for the network, as some of the existing SN-CIRB Reliance Agreements were set to expire on July 31, 2023. Additionally, significant changes occurred in guidance related to using a sIRB for multi-site clinical trials and in the operations of the SN-CIRB since the execution of the original StrokeNet Reliance Agreement.

One of the most notable developments during this period was the launch of SMART IRB (Streamlined, Multi-site, Accelerated Resources for Trials IRB Reliance platform) in 2016, funded by the National Center for Advancing Translational Sciences https://smartirb.org/. SMART IRB provides an IRB reliance agreement, along with supportive tools and resources, to streamline and harmonize the sIRB process [9]. Over 1300 institutions have joined SMART IRB and used its master agreement to facilitate IRB reliance for multi-site research. Once it is signed, institutions may use the agreement either to serve as the sIRB for other parties or to rely on another party’s sIRB. Negotiating any agreement terms is unnecessary when starting a new study. Most research institutions can accept it.

Given the widespread acceptance of the SMART IRB Agreement and the many StrokeNet sites that had already signed it, we decided to encourage its use within StrokeNet to streamline reliance processes and enhance efficiency across the network. This approach was further supported by the successful transition of the Collaborative Pediatric Critical Care Research Network from site-specific agreements to the SMART IRB Agreement [10]. The UC HRPP Reliance team started with a list of 850 sites. We removed duplicate Federal Wide Assurances and sites where the UC IRB does not serve as the reviewing IRB, leaving us with 371 sites to contact. Of these, 247 sites had already signed the SMART IRB Agreement Version 2.0, further supporting the transition to this standardized reliance framework. Participating institutions that had already signed the SMART IRB agreement received a SMART IRB Letter of Acknowledgment to document that the SMART IRB agreement would be used for StrokeNet studies conducted at their institutions. Flexible components of the SMART IRB Agreement, as implemented by the SN-CIRB, were addressed in the letter. Flexible components are areas of responsibility among studies that can vary and must be documented.

The flexible components implemented by the SN-CIRB included applying NIH StrokeNet and UC CIRB SOPs; required reporting to regulatory and funding agencies; requiring participating sites to conduct COI reviews and develop management plans when needed; and conducting IRB-initiated audits or investigations on a case-by-case basis. The flexible components also assign the SN-CIRB the responsibility for HIPAA determinations required for the Relying Institution to use or disclose Protected Health Information. If an authorization is needed, the Reviewing IRB will determine the form of the authorization. If an alteration or waiver of authorization is requested, the SN-CIRB will perform the analysis and is responsible for granting the waiver or alteration. The SN-CIRB decided to take on these responsibilities based on the NeuroNEXT process and to streamline the reliance process for relying sites.

Non-SMART IRB institutions were encouraged to sign onto the SMART IRB agreement. Sites unable or unwilling to join SMART IRB received a standard UC CIRB Reliance Agreement. The key difference between the standard UC CIRB Reliance Agreement and the SMART IRB Agreement is that the UC Agreement does not require an HRPP quality assessment, which is required under the SMART IRB Agreement when an organization has an internal IRB(s). Since a number of StrokeNet sites, even some with an internal IRB, have not undergone a formal HRPP quality assessment, this requirement was omitted from the UC Agreement. In such cases, we instead request copies of any local human research policies to confirm that the CPS has adequate resources and procedures to oversee StrokeNet studies. It is unclear why sites without an IRB did not sign the SMART IRB agreement. Perhaps it was due to a misunderstanding of the SMART IRB agreement requirements for HRPP quality assessment. The components of the reliance agreement remained consistent with the previously executed StrokeNet reliance agreement. Site HRPPs selected applicable studies and signed and returned the executed agreement. Of the 371 sites, 211 agreed to use the SMART IRB agreement to document reliance on the SN-CIRB for StrokeNet studies (Table 2). Forty-three sites were unwilling or unable to join SMART IRB. The remaining 117 sites decided not to sign a new agreement to continue to use the SN-CIRB. The main reason for not signing was that the sites were not participating in any StrokeNet studies. Surprisingly, it took an average of over 100 days for sites to approve either the SMART IRB Agreement or the UC IRB Reliance Agreement. This was significantly longer than the time required to execute the initial agreements. We believe the extended timeline resulted from the new agreement requests being sent out well before the initial agreement expiration dates. Without a sense of urgency, sites did not prioritize the approval process. Also, many of the listed points of contact at the sites were no longer correct, and multiple notifications had to be sent to identify the proper contact. Importantly, no initial agreements expired before a new agreement was in place. Because the NCC had already established a process for documenting reliance and some sites elected not to use the SMART IRB Agreement, the SMART IRB Online Reliance System was not utilized. This approach ensured consistency in documentation and procedures across all participating sites. In addition, because the online reliance system records reliance on a per-trial basis rather than at the network level, and given the large number of participating sites, the decision was made not to require each site to document reliance individually for every trial.

**Table 2.** StrokeNet Reliance Renewal Project 2023

StrokeNet Reliance Renewal Project 2023	
Total Relying Sites Contacted	371
Reliance Agreements obtained	254
SMART Reliance Agreements obtained	211
UC Reliance Agreements obtained	43
Total Reliance Agreements not obtained	117
SMART participating sites not renewed	42
Other sites not renewed	75
Reasons for not renewing	117
Inactive*	76
Consolidated under a different FWA	13
A different institution signs the Reliance Agreement	20
RA on file has not expired	7
Pending (i.e., site is in the process of closing)	1
Site transitions to SMART during the renewal project	17
Average number of days to obtain agreement	
SMART Reliance Agreement	121.2
UC Reliance Agreement	112.3

*Sites not currently participating in any StrokeNet studies.

## Management of the StrokeNet Central IRB

The UC HRPP and the NCC jointly manage the SN-CIRB process, with reportable event information collected through StrokeNet’s clinical trial management system, WebDCU™, which is managed by the NDMC. As outlined below, the NCC utilizes the UC HRPP centralized electronic system, the Research Administrative Portal (RAP), to submit materials for SN-CIRB review. The SN-CIRB likewise uses RAP to communicate determinations, correspondence, and other review-related documents back to the NCC.

An NCC Project Manager is assigned to each study to oversee its administrative management, including coordinating document submissions to the SN-CIRB, responding to queries, and disseminating approved materials. Regulatory Specialists at the NCC also help with managing the exchange of information. The StrokeNet award funds a dedicated SN-CIRB Liaison position within the UC HRPP. This individual serves as the primary point of contact between SN-CIRB, the UC HRPP, the NCC, and the NDMC. The liaison facilitates communication among these entities, assists with the CIRB review of StrokeNet studies, and supports the submission of studies to the UC HRPP. The NCC Project Managers and SN-CIRB Liaison maintain direct communication to facilitate the efficient exchange of information between the SN-CIRB and study investigators.

The lead study site must provide protocol-level information applicable to all CPS, and each CPS must provide site-level details relevant only to its own site in a thorough and timely manner to the NCC, to ensure the process works efficiently for the NCC. Clear and timely communication among all participants is essential for effective coordination. Each group has distinct responsibilities that contribute to the overall efficiency of the process.

Communication between the NCC and research sites is conducted primarily via email. NCC program managers are responsible for entering protocol information into RAP and distributing the SN-CIRB-approved materials to participating sites. The UC-HRPP determined that assigning this responsibility to the program managers was the most efficient approach, as it eliminates the need for investigators to obtain UC system access and training. Utilizing program managers who are experienced with RAP promotes accuracy, streamlines submissions, and minimizes the need for clarifications before SN-CIRB review. Since the NCC manages communications with the CPS outside of RAP, the Huron IRB Exchange is not used to facilitate communication among sites and document reliance https://huronirbexchange.com/.

*The SN-CIRB and the NCC have jointly developed several NIH StrokeNet Standard Operating Procedures (SOPs) to guide the management and oversight of the SN-CIRB. These SOPs define the respective responsibilities of the SN-CIRB, research teams, and relying institutions. Use of these SOPs is part of the flexible components of the reliance agreements implemented by the SN-CIRB. The SOPs are available on the NIH StrokeNet website to allow ongoing access for participating sites. Updates and revisions to the SOPs are communicated and reviewed with sites during monthly webinars. Additionally, study-specific office hours are offered to allow sites to consult with NCC Project Managers regarding protocol implementation and interpretation of applicable SOPs.*

NIH StrokeNet Network Standard Operating Procedure: ADM 11 CIRB Reliance and Approvals. This SOP outlines the process for institutions engaged in NIH StrokeNet-affiliated research to transfer human subjects review to the SN-CIRB or an independent IRB. The SOP includes the use of an independent IRB as an alternative to ensure continuity in review if the SN-CIRB is unable to provide a timely review due to logistical constraints. Since SN-CIRB is based at a public university, it can be challenging to hire new staff to address increased workloads quickly.

An independent IRB currently oversees four primary StrokeNet studies and one ancillary study. Its administrative review process follows the IRB’s internal procedures. The independent IRB’s role in reviewing StrokeNet research is outlined in the StrokeNet SOPs governing single IRB oversight.

**Figure 1.**
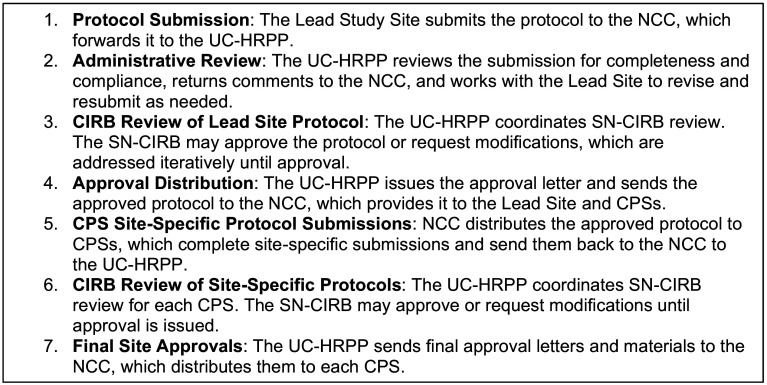
Overview of the protocol review and approval process.

**Figure 2.**
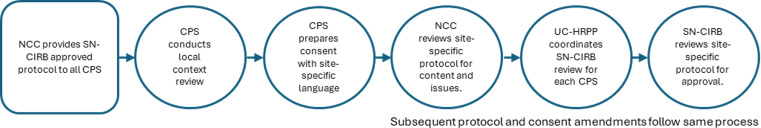
Clinical Performance Site (CPS) approval process.

This SOP provides a comprehensive overview of protocol review procedures and outlines the flow of information between the Relying Institution and the IRBs throughout the protocol lifecycle (Figure 1). As described in SOP ADM 11, the SN-CIRB is responsible for the regulatory review of StrokeNet protocols to ensure they meet all applicable federal human subjects protection regulations. SN-CIRB defines the study protocol as the comprehensive, written description of the research project. It includes a model informed consent document and any additional forms required by the SN-CIRB, as determined on a trial-by-trial basis. Study protocols that present greater than minimal risk are reviewed and approved by the SN-CIRB at a convened board meeting. Study protocols that involve no more than minimal risk may be eligible for expedited review. After SN-CIRB approves the study protocol, the NCC collaborates with participating performance sites to facilitate the implementation of the study protocol at each site. CPS must obtain SN-CIRB approval to participate, typically through an expedited review process. This same approach is used to review and approve protocol modifications and continuing reviews (Figure 2).

NIH StrokeNet Network Standard Operating Procedure: ADM 02 Reporting Conflict of Interest and Financial Disclosures This SOP describes how NIH StrokeNet ensures compliance with HHS and FDA financial conflict of interest (COI) regulations. It defines the SN-CIRB’s role in identifying and managing trial-specific COIs, particularly those involving the protocol and site Principal Investigators (PIs). SN-CIRB collaborates with relying sites to assess and manage PI COIs, protecting research participants and maintaining study integrity.

Relying sites are responsible for identifying and managing COIs for other site investigators and must report any such conflicts to the SN-CIRB. While the SN-CIRB defers to institutional COI policies for managing these cases, it may implement additional protocol-specific safeguards when necessary to ensure subject protection and research credibility. The UC-COI Officer assists the SN-CIRB in the identification and management of COIs.

NIH StrokeNet Network Standard Operating Procedure: ADM 12 Central Institutional Review Board (CIRB) Reporting. This SOP defines the types of reports that must be submitted to the SN-CIRB, outlines the reporting timeframes, and describes the process for these submissions, ensuring consistency in reporting across the network. Under the terms of the Reliance Agreement, the SN-CIRB is responsible for reviewing required reports, which may include unanticipated problems involving risks to subjects or others (UPIRTSO), protocol noncompliance issues, subject injuries, subject complaints, as well as protocol violations and deviations involving increased risk. When reviewing these reports, the SN-CIRB determines whether the event constitutes a UPIRTSO or rises to the level of serious or continuing noncompliance with applicable laws, regulations, or SN-CIRB requirements and determinations.

## Revisions to the administrative process to improve efficiency

Since its establishment, SN-CIRB, in collaboration with the UC HRPP and the NCC, has implemented a series of process improvements to reduce administrative burden while maintaining rigorous protections for research participants. These refinements are informed by ongoing evaluation and lessons learned from protocol reviews.

### Revisions to StrokeNet SN-CIRB forms

The NCC and UC HRPP revised the StrokeNet Local Site Context, Performance Site Application, and HIPAA Waiver of Authorization forms to address common areas of confusion that require frequent follow-up with sites. Questions were restructured to better reflect local site practices, thereby enhancing the accuracy and completeness of submissions. For example, checkboxes with response options were added to some forms to reduce reliance on free-text answers. The forms are reviewed and updated as needed to address the requirements of specific protocols.

### Workflow optimization

In 2019, SN-CIRB reviews were integrated into the general HRPP workflow, transitioning from a dedicated SN-CIRB team to a model where all UC HRPP coordinators are trained to manage StrokeNet reviews. This cross-training enhanced operational flexibility and efficiency while maintaining the quality of reviews.

### New electronic IRB system implementation

The initial version of the Electronic IRB System did not include a method for managing the lead study and the sites separately while also keeping them linked. The lead and study site records were entered as independent records within the system. Unique labeling was used to provide a visual link between records, and additional tracking was maintained outside the system as a snapshot of the overall study status, which was inefficient to maintain.

In 2019, the UC HRPP implemented RAP, built on a commercial, centralized electronic IRB platform, “Huron IRB.” RAP removed the need for unique labeling and additional tracking. The SmartForm and workflow were revised to identify multi-site projects and integrate management of both shared and separate review processes for the lead study and sites. This streamlined SN-CIRB operations by enabling sites to be linked to the lead study site, enhancing efficiency and communication across all participating sites. NCC Program Managers and Regulatory Specialists submit site-specific materials through RAP for SN-CIRB review, while UC HRPP Coordinators manage the review process within the system. Once the review is complete, the coordinators upload approved documents to RAP, allowing Program Managers and Regulatory Specialists to distribute them to sites.

### Key personnel review revisions

The HRPP revised its process for SN-CIRB approval of key personnel in alignment with the SMART IRB Harmonization Guidance on the Process for Review of PI and non-PI personnel for Multi-Site Studies. Sites now assume responsibility for verifying the qualifications of local investigators and staff and ensuring financial COIs are appropriately managed. SN-CIRB review is now limited to approval of Principal Investigators (PIs) and any staff with positive disclosures. This change substantially reduced the administrative workload for the HRPP, SN-CIRB, and NCC. For example, in a sampling of 10 ARCADIA sites, 29 of 48 modifications approved by the SN-CIRB were for site personnel changes before the implementation of this change.

### Continuing review process improvements

The SN-CIRB implemented significant revisions to the continuing review process, informed by the SMART IRB Harmonization Guidance SINGLE IRB CONTINUING REVIEW PROCESS: Recommendations for Harmonization. Changes include no longer requiring a copy of each site’s most recent signed informed consent document (ICD) to be submitted as part of the continuing review. This step was previously used to document that the correct version of ICD was being used. However, the SN-CIRB determined that the NCC process for distributing the ICD ensures that the currently approved ICD is used at the sites. Additionally, all signed ICDs are also monitored by the NDMC, and their versions are verified. Eliminating the review of the most recently signed ICD from the sites has decreased the number of documents and time required for sites gathering them, as well as time spent reviewing the versions by the IRB Coordinators with the pre-review of the submission documents and clarifications if sites did not properly redact the participant’s names and signatures.

Event reporting is now categorized into three groups: those requiring prompt reporting, those reportable at continuing review, and those not requiring SN-CIRB reporting. This structure aligns with the SMART IRB REPORTABLE EVENTS: Recommendations for Investigator-initiated Multisite Studies. Protocol deviations that are captured in WebDCU^TM^ but are not considered unanticipated or serious adverse events no longer need to be reported to the SN-CIRB at the time of continuing review. This updated approach was designed to reduce administrative burden for study teams, the NCC, and the SN-CIRB, to streamline the preparation, submission, and review of continuing reviews without increasing risk to participants. The SN-CIRB concluded that, with clearer event reporting criteria, such deviations are highly unlikely to constitute serious or continuing noncompliance or a UPIRTSO. In addition, the lead investigator and the DSMB remain responsible for ongoing monitoring of these events and for reporting any that, individually or in aggregate, may represent apparent serious or continuing noncompliance or UPIRTSOs.

### Central electronic informed consent (eConsent) process

The SN-CIRB collaborated with the NCC to develop the NIH StrokeNet Network Standard Operating Procedure ADM 24 Central Electronic Informed Consent Process. This SOP provides a framework for using a centrally managed eConsent platform (Research Electronic Data Capture, also known as REDCap, Nashville, TN). The SOP is consistent with FDA and OHRP guidance on electronic informed consent and promotes standardization across StrokeNet studies. The process complies with 21 CFR Part 11 through REDCap’s technical features combined with the StrokeNet policies, training, validation, and documentation practices. The StrokeNet eConsent process has been widely adopted among sites utilizing electronic consent. In a random sample of 30 SISTER research sites, 27 reported using eConsent, and 25 used the StrokeNet eConsent process. The process is associated with higher enrollment at individual sites, increased use of remote consent, and better adherence to consent documentation requirements compared to the use of a paper ICD [11].

### SN-CIRB -DSMB joint operating procedure

Recognizing the overlap in IRB and DSMB oversight responsibilities when an sIRB oversees a multisite study, the SN-CIRB, DSMB, and NCC developed the NIH StrokeNet Network Standard Operating Procedure ADM 25 StrokeNet CIRB and DSMB Joint Operating Procedure. This SOP defines the roles, responsibilities, and communication pathways between the SN-CIRB and DSMB during the study review and oversight process. It is the first formalized mechanism, to our knowledge, for direct interaction between a DSMB and an IRB, designed to prevent conflicting decisions and ensure participant safety and scientific integrity. The SOP has since informed similar efforts in other networks, including the Strategies to Innovate Emergency Care Clinical Trials Network.

### Budget support of SN-CIRB

Historically, IRB review costs for NIH-funded studies have been covered by the Facilities and Administration (F&A) or indirect costs as discussed in the SMART IRB Harmonization Guidance Fees and Costing Models under the NIH sIRB Policy. However, with the implementation of the sIRB mandate, NIH guidance indicates that secondary activities related to sIRB of multisite studies may be considered allowable costs. Essentially, any secondary review activities that exceed the primary work required to review a protocol at a site may be considered allowable costs. The NIH determined that the cost of operating the SN-CIRB would go beyond the standard operating costs of the IRB and allowed the cost of the SN-CIRB review to be included in the direct costs of the initial StrokeNet award. Subsequently, the cost of the SN-CIRB review is now included in the individual study budgets.

## Discussion

The success of the SN-CIRB demonstrates that an Academic Health Center-based IRB can effectively function as a sIRB for a large clinical trial network. While the SN-CIRB protocol approval times may be longer than those typically seen at independent IRBs, the SN-CIRB adds value to NIH StrokeNet by maintaining open communication with the NCC, lead investigators, and study teams and providing individualized guidance from experienced IRB professionals throughout the study life cycle. This efficiency is the result of strong collaboration among the NDMC, the NCC, the UC HRPP, and the SN-CIRB. These groups prioritize reducing the administrative burden while maintaining robust participant protections. Lead investigators and research sites must also understand their roles in the process. Research sites must recognize that the SN-CIRB serves as the IRB of record for their site; therefore, their local IRBs should not conduct a regulatory review of the studies. While research sites have other responsibilities during the process, these should be managed by a local office, such as an HRPP, rather than overseen by the local IRB.

A key factor in ensuring the smooth operation of the SN-CIRB is the CIRB Liaison, who oversees all submissions and serves as the primary point of contact for the NCC and participating sites.

The SN-CIRB Chair works directly with lead investigators during protocol development to identify and address potential issues related to risk–benefit assessment and IRB approval criteria. Examples of common problems include justification for including vulnerable populations, variations in protocol-mandated standard-of-care procedures across sites and plans for future use of data and biospecimens.

Another approach to addressing this issue is to establish a sIRB consultation resource. The Utah Trial Innovation Center implemented such a resource to support study teams in developing multisite, cooperative research proposals before submission for funding [12].

Several barriers limit the efficiency and enthusiasm of the HRPP/IRB community regarding the sIRB process [13,14]. One challenge, as discussed above, is the tendency of some relying institutions to conduct unnecessary, duplicative reviews. Another approach to addressing this issue is to establish a sIRB consultation resource.

The SMART IRB Harmonization Guidance on Institution v. IRB Responsibilities identifies the responsibilities that relying institutions maintain when relying on an sIRB for oversight of studies at their institutions. To address this issue on StrokeNet studies, the NCC and SN-CIRB distributed the StrokeNet Memorandum for Local Site Context Review at Relying Institutions, which reinforces that while relying institutions retain responsibility for certain aspects of local study oversight, such as site-specific considerations, local ancillary requirements, and ongoing oversight of research, their local IRBs should not conduct a full regulatory approval review of the protocol.

Another challenge is the variability in how reviewing IRBs conduct their reliance reviews, which makes it difficult for relying institutions and study teams working with multiple IRBs to comply with differing requirements. To address this issue, SN-CIRB has incorporated SMART IRB harmonization guidance into several of its processes, including continuing review procedures and the approval of key personnel. Future evaluation of the SN-CIRB process will include a detailed analysis of CPS approval times to identify additional areas for improvement to increase the efficiency of the process.

While challenges to the efficient operation of CIRBs for clinical research networks persist, the experience of the SN-CIRB and others shows that with continued innovation and collaboration, centralized IRB review can be further streamlined and optimized to support the future of multi-site research.
